# Is routine magnetic resonance imaging necessary in patients with clinically diagnosed frozen shoulder? Utility of magnetic resonance imaging in frozen shoulder

**DOI:** 10.1016/j.jseint.2022.05.009

**Published:** 2022-06-11

**Authors:** Dimitris Dimitriou, Elin Winkler, Christoph Zindel, Florian Grubhofer, Karl Wieser, Samy Bouaicha

**Affiliations:** Balgrist University Hospital, Orthopaedic Department, University of Zurich, Zurich, Switzerland

**Keywords:** Frozen shoulder, Adhesive capsulitis, Shoulder pain, MRI, Cost-effectiveness, Rotator cuff tears

## Abstract

**Background:**

Shoulder magnetic resonance imaging (MRI) is commonly performed in patients with frozen shoulder (FS). However, the necessity of MRI and its diagnostic value is questionable. Therefore, the purpose of the present study was to clarify whether routine MRI could identify additional shoulder pathologies not previously suspected in the clinical examination and if any change in the treatment plan based on these additional MRI findings in FS patients was observed.

**Materials and methods:**

The medical records of all patients who presented in our outpatient clinic with a diagnosis of FS from January 2017 to December 2018 were retrospectively reviewed. Patient demographics, the number of patients who received a shoulder MRI, changes in the diagnosis or identification of structural shoulder pathologies following MRI examination (if performed), as well as any alternation in the initially suggested treatment plan were recorded.

**Results:**

A total of 609 patients (male: 241, female: 368) diagnosed with an FS and an average age of 52 ± 10 (range: 18 to 81) years were identified. In 403 of the 609 patients (66%), a shoulder MRI was performed. An additional structural shoulder pathology was identified in 89 of 403 (22%) patients following the shoulder MRI, mostly rotator cuff tears (partial: 46/403 [11.4%], full-thickness: 30/403 [7.4%], rerupture following reconstruction: 10/403 [2.5%]) and labrum tears (3/403 [0.7%]). At minimum 2-year follow-up, 11 of 403 (2.7%) patients were treated surgically for the additional pathology identified on the MRI scan consisting of an arthroscopic rotator cuff reconstruction in 10 patients and a labrum refixation in one patient. Five of the 609 (0.8%) patients were treated for refractory FS by arthroscopic capsulotomy.

**Conclusions:**

Although additional pathologies were identified in 22% of the patients, a change in treatment plan due to the MRI findings was only observed in 2.7% (37 MRIs needed to identify 1 patient with FS requiring surgery for the additional MRI findings). Therefore, routine use of shoulder MRI scans in patients with FS but without suspicion of an additional pathology may not be indicated.

Frozen shoulder (FS), sometimes also referred to adhesive capsulitis, is a debilitating condition characterized by an insidious onset of shoulder pain, progressive stiffness, and significant restriction of range of motion (ROM) affecting up to 5% of the general population.^8^^,^^9^ FS can be classified as primary (idiopathic or secondary), which can be further divided into intrinsic (shoulder pathologies, posttraumatic or postsurgical), extrinsic, and systemic (thyroid disease, diabetes mellitus).^25^ Although FS is a self-limited condition with a satisfying recovery in the majority of the cases, the average duration of symptoms is 15 months (range: 12 to 30)^23^ and is associated with a high socioeconomic burden.^2^ The diagnosis of FS can be challenging because it remains a diagnosis of exclusion and is based mainly on clinical examination. The only additional imaging suggested is a plain shoulder radiograph to rule out other possible pathologies causing a limited ROM such as osteoarthritis, fracture, and chronic shoulder dislocation.^12^

Magnetic resonance imaging (MRI) with or without intraarticular injection of dye is regarded as the reference standard for rotator cuff pathologies^11^ due to its excellent contrast resolution, optimal soft-tissue visualization, and multiplane scanning function.^18^ MRI has demonstrated excellent diagnostic accuracy in detecting rotator cuff tears,^13^ shoulder osteoarthritis,^19^ labrum tears,^1^ as well as acromioclavicular joint pathology.^22^ However, the prevalence of pathologic findings in shoulder MRIs in asymptomatic patients is high and leads to irrelevant diagnoses for patients with consecutive overtreatment.^6^^,^^7^^,^^20^ Recent literature found several MRI findings associated with FS^15^^,^^24^ such as thickening of the coracohumeral ligament and rotator cuff interval,^10^ decreased volume of the axillary recess, and obliteration of the subcoracoid fat triangle.^4^

With the increasing availability of MRI comes a large number of unnecessary shoulder MRI scans, ranging from 41% to 56%.^5^^,^^21^ Given the high cost of an MRI scan in Switzerland (900 CHF [$954] for an MRI and 1200 CHF [$1272] for an MRI-arthrogram) and also worldwide (£153 and £272 in the United Kingdom and $2033 and $2339 in the United States for an MRI and MRI-arthrogram, respectively^17^), the necessity of MRI in the diagnosis of FS is not yet justified.

Therefore, the purpose of the present study was to report the frequency of performed shoulder MRI scans in patients with a clinically diagnosed FS, the incidence of an additional shoulder pathology not previously suspected in the clinical examination/conventional shoulder radiograph, and any change in the treatment regimen based on the additional MRI findings.

## Materials and methods

### Study design and patient selection

The study was approved by our institution's internal review board as well as by the local ethical committee and was entirely conducted at the authors' institution. The medical records of all the patients who presented in our outpatient clinic from January 2017 to December 2018 were retrospectively reviewed. The clinical examination described in the charts was performed or supervised by an experienced shoulder surgeon at our institution.

### Inclusion and exclusion criteria

The clinical criteria for the diagnosis of FS were a painful and stiff shoulder presenting for at least 4 weeks, with a restricted passive external rotation of <20°.^14^ Patients older than 18 years who presented in our outpatient clinic with the diagnosis “adhesive capsulitis” or “frozen shoulder” on their medical report were included in the study. They were further divided into subgroups based on the etiology such as idiopathic, posttraumatic, and postoperative FS. Exclusion criteria were evidence of glenohumeral arthritis on plain x-ray scans, patients on whom a shoulder arthroplasty was performed, a history of shoulder infection or neurologic disease (eg, Parkinson, cerebrovascular disease), all capable of limiting the ROM of the shoulder. Furthermore, patients with contraindications for an MRI scan such as claustrophobia or pacemaker were excluded.

### Data collected

Patient demographics such as age, gender, and cause of FS (idiopathic, posttraumatic, postoperative) were collected. Furthermore, frequency of MRI performed, the diagnosis on the first medical report, as well as changes thereof and identification of additional shoulder pathologies following a shoulder MRI (if performed), alternations in the suggested treatment plan, radiology report, who requested the MRI scan (primary physician or orthopedic surgeon), and the time from onset of symptoms to MRI scan were analyzed.

### Statistical analysis

Descriptive statistics used frequencies and percentages to present the data. The patients were divided into 2 groups depending on if an MRI scan was performed. A 2-tailed t-test was used to compare the demographics between the groups and the time from the beginning of symptoms to the MRI scan. The chi-square test was performed to compare the observed frequencies. The number of MRI scans needed to identify a patient requiring surgery for the additional shoulder pathology was defined as the total number of MRI scans performed divided by the number of patients treated surgically for the additional MRI finding. All the statistical analyses were performed using SPSS version 23 software (SPSS Inc., Armonk, NY, USA).

## Results

A total of 609 patients (male: 241, female: 368) diagnosed with FS and of an average age of 52 ± 10 (range: 18-81) years were included in the study. Idiopathic FS was diagnosed in 240 of 609 (40%), posttraumatic in 183 of 609 (30%), and postoperative in 186 of 609 (30%) patients (Table I).

**Table I:** Patient characteristics.

Characteristics	MRI performed (n = 403)	MRI not performed (n = 206)	Significance (*P* value)
Age, mean ± SD (range), yr	52.7 ± 9.5 (19, 80)	52 ± 11.4 (18, 81)	n.s.
Female gender, %	59%	63%	n.s.
Right shoulder, %	48%	49%	n.s.
Cause of adhesive capsulitis			
Idiopathic	40%	40%	n.s.
Posttraumatic	34%	21%	<.01
Postoperative	26%	39%	<.01

*MRI*, magnetic resonance imaging; *n.s.*, not significant; *SD*, standard deviation.

In 403 of the total 609 patients (66%), a shoulder MRI was performed (Table I). There was no significant difference between the groups with or without an MRI scan except for MRI scans being performed more often in posttraumatic FS (34% vs. 21%, *P* < .01) and less commonly in postoperative FS (26% vs. 39%, *P* < .01). Intraarticular contrast dye was used in 345 of the 403 (85.6%) MRIs. The MRI was requested by the primary care physician in 208 of the 403 (51.6%) cases at an average of 4.8 ± 5.1 (range: 0-42) months, whereas a shoulder surgeon requested the MRI in 195 (48.4%) at an average of 10.9 ± 10.3 (range: 4-108) months following the onset of symptoms.

An additional shoulder pathology was identified in 89 of the 403 (22%) patients following the shoulder MRI scan. These consisted mostly of rotator cuff tears, partial in 46 (11.4%), full-thickness in 30 (7.4%), and rerupture following reconstruction in 10 of the 403 cases (2.5%) as well as labrum tears in 3 (0.7%). In 34 of the 89 (38.2%) cases, the additional shoulder pathology was associated with a trauma; in 15 (16.9%), with surgery; and in 40 (44.9%), with idiopathic FS. In the nontraumatic, nonoperative group, there was only a slightly higher risk for additional findings on MRI scans with increasing age (odds ratio 1.01, 95% confidence interval 1.006-1.106). At the minimum follow-up of 2 years, 11 of the 403 (2.7%) patients were treated surgically for the additional structural pathology identified on the MRI scan, consisting of an arthroscopic rotator cuff reconstruction in 10 patients and a labrum refixation in 1 patient. According to these results, 37 MRI scans are needed to identify 1 patient with FS requiring surgery for the additional MRI findings. Another 5 of 609 (0.8%) patients clinically diagnosed with FS were treated with an arthroscopic capsulotomy.

## Discussion

FS is a debilitating shoulder pathology which is usually diagnosed based on clinical examination. Different studies have reported a prevalence of FS ranging from 5% to 33% depending on the etiology.^8^^,^^9^ With increasing availability, the use of MRI shoulder scans has constantly increased when dealing with this pathology. In the present study, an MRI scan was performed in 66% of patients with a clinically diagnosed FS from January 2017 to December 2018, resulting in additional unnecessary health costs per patient. In total, an additional shoulder pathology was identified in 22%, and in 2.7%, this pathology resulted in a surgical treatment. According to these results, 37 MRI scans are needed to identify 1 patient with FS requiring surgery for the additional MRI findings.

There are several arguments righteously questioning the necessity of a expensive MRI scan when diagnosing FS in patients. First, FS can be diagnosed solely with a proper assessment of the patient's history including risk factors (diabetes, thyroid gland pathologies) and clinical examination in combination with an x-ray to rule out pathologies that may also cause a limited ROM such as osteoarthritis, fracture, or a chronic shoulder dislocation.^12^ Additionally, even though symptoms may, on average, persist up to 15-18 months, FS is known to be a self-limiting disease,^2^^,^^23^ thereby reducing the need for an MRI shoulder scan when waiting long enough for the symptoms to resolve. A recently published study also suggested that x-ray imaging may aid in diagnosing FS by measuring humeral head migration.^3^ Furthermore, patients with FS demonstrate similar rates of rotator cuff tears as asymptomatic patients, with a prevalence of 15.2% in the sixth decade of life. The results of our study also showed a slightly higher risk for additional shoulder pathologies on MRI scans with increasing age in idiopathic FS. Furthermore, Minagawa et al^16^ reported that 65.3% of all rotator cuff tears tend to be asymptomatic. Another study reported up to 34% of asymptomatic shoulders have rotator cuff tears on shoulder MRI scans,^21^ thereby questioning if the additional pathologies on MRI scans are responsible for the symptoms in FS patients. Moreover, the additional structural but potentially asymptomatic lesion might mislead the physician to an unnecessary surgical overtreatment.

In the present study, an additional shoulder pathology was identified in 22% of the patients; however, only 11 of the 403 patients (2.7%) had to be treated surgically. The challenge, especially in posttraumatic or postoperative FS, remains in selecting the individuals in need of an MRI scan to detect additional damage, represented by the 2.7% (11/403) in our study, in whom the treatment plan changed based on the MRI findings. However, this also means that in 392 of the 403 (97.3%) patients, the MRI scan showed no clinically relevant additional shoulder pathology. When critically interpreting these results, although a full-thickness rotator cuff tear was diagnosed in 7.4% of patients, the rest of the 392 patients received an unnecessary MRI scan to exclude an additional relevant pathology causing similar symptoms. Freeman et al^5^ analyzed the appropriateness of an MRI scan for different shoulder pathologies and concluded only 56% of scans falling into that category. However, shoulder specialists deem MRI shoulder scan more appropriate than other specialties (70% vs. 38%).^5^ In the present study, about the same amount of MRI scans were ordered by specialists and primary care physicians (48.4% vs. 51.6%); however, the duration from onset of symptoms to the performed MRI scan was significantly higher when requested by the specialist (4.8 months vs. 10.9 months, *P* < .01). In posttraumatic FS, MRI scans were performed significantly more often, possibly to exclude acute injuries. Patients with postoperative FS less frequently received an MRI scan probably since some limited ROM is part of the normal healing process.

The present study should be interpreted in light of its potential limitations. The most obvious drawback was the retrospective study design. However, due to the detailed documentation at a university hospital setting, the necessary patient data were available for full analysis. Furthermore, some general practitioners might not be as familiar with the different shoulder pathologies, and as a result, some of these might not have been recognized in the clinical examination and could have been referred with the wrong suspected diagnosis. Nonetheless, the diagnosis was later adjusted and documented correctly by an experienced shoulder specialist.

To the best knowledge of the authors, no current literature investigating the necessity of shoulder MRI in patients with FS in terms of diagnosing additional structural shoulder pathologies and resulting changes in treatment plan exists. The results of the present study are of clinical relevance as they could improve diagnostic protocols and reduce the use of unnecessary MRIs in patients with FS.

## Conclusion

Although additional pathologies were identified in 22% of patients, with 7.4% showing a full-thickness tear, a change in treatment plan due to the MRI findings was observed in only 2.7% of the patients. Given these numbers 37 MRIs are needed to identify 1 patient with FS requiring surgery for the additional MRI findings. Therefore, routine use of shoulder MRI scans in patients with FS but without suspicion of an additional pathology may not be indicated and can create unnecessary health-care costs. The results of the present study are of clinical relevance as they improve diagnostic protocols and reduce the use of unnecessary MRI scans in patients with FS. We have strongly reduced the routine use of MRI in FS patients, limiting its indication to patients with unusual, prolonged symptoms and/or clinical symptoms suggesting an underlying structural shoulder pathology.

## Disclaimers:

Funding: No funding was disclosed by the authors.

Conflicts of interest: The authors, their immediate families, and any research foundation with which they are affiliated have not received any financial payments or other benefits from any commercial entity related to the subject of this article.
